# Protocol for fixation and staining of *Drosophila melanogaster* embryos optimized for DNA-PAINT nanoscopy of adherens junction proteins

**DOI:** 10.1016/j.xpro.2026.104830

**Published:** 2026-09-12

**Authors:** Neelima Sharma, Matthias Häring, Jennifer H. Richens, Daniel St Johnston, Jörg Großhans, Deqing Kong

**Affiliations:** 1 Department of Biology, University of Marburg, Marburg, Germany; 2 Göttingen Campus Institute for Dynamics of Biological Networks (CIDBN), University of Göttingen, Göttingen, Germany; 3 The Gurdon Institute and Department of Genetics, University of Cambridge, Cambridge, UK; 4 Key Laboratory of Evolution & Marine Biodiversity (Ministry of Education) and Institute of Evolution & Marine Biodiversity, Ocean University of China, Qingdao, China

**Keywords:** Cell Biology, Microscopy, Model Organisms

## Abstract

Cadherin-catenin based adherens junctions are dynamic protein complexes that mediate homophilic cell-cell adhesion, strengthen cell-cell contact, and maintain cell polarity. Here, we present a protocol to prepare *Drosophila* embryo samples for DNA-PAINT nanoscopy to resolve nanoscale E-cadherin clusters at junctions. We describe steps for embryo collection, fixation, immunostaining, and mounting onto glass-bottom chambered slides to prepare samples suitable for DNA-PAINT nanoscopy. DNA-PAINT microscopy provides insight into the nanoscopic clustering and arrangement of E-cadherin complexes in fixed tissue.

For complete details on the use and execution of this protocol, please refer to Häring et al.^1^

## Highlights

• Protocol to prepare *Drosophila melanogaster* embryos for DNA-PAINT nanoscopy
• Instructions for raising flies and collecting gastrulating embryos
• Steps for fixation and immunostaining of embryos, optimized for DNA-PAINT imaging
• Guidance on quantitative correlation analysis of super-resolution junction images

## Abstract (editor highlights)

Publisher’s note: Undertaking any experimental protocol requires adherence to local institutional guidelines for laboratory safety and ethics.

## Graphical abstract

**Figure:**
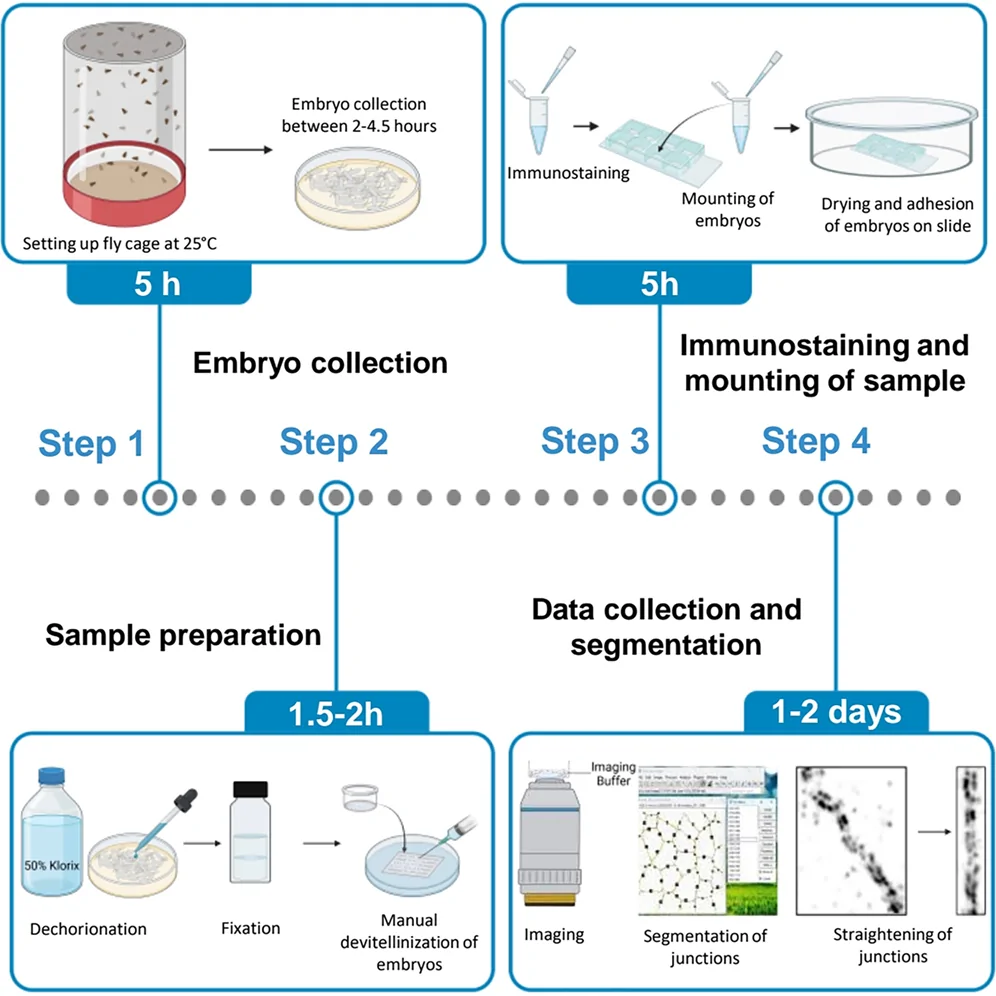

## Before you begin

Junction proteins physically integrate individual cells into tissue and serve as permeability barriers. Adherens junctions (AJs) in epithelial cells are comprised of an E-cadherin-β-catenin-α-catenin trimeric complex, which binds to the intracellular actomyosin cortex as well as to an E-cadherin molecule of the juxtaposed cell via its extracellular domain and thus physically links cells and controls their arrangement in a regular single- or multi-layered epidermis structure.^2^^,^^3^ Beyond their structural role, AJs confer mechanical cell-cell communication by connecting the neighboring actomyosin cortex and play an important role in mechanotransduction.^4^

A key feature of AJ organization is E-cadherin molecules organized into discrete nanoclusters required for stability of cell-cell contacts and serving as platforms for mechanosensing.^2^^,^^5^ AJs are dynamic and increased mechanical tension across junctions triggers conformational changes in α-catenin, exposing binding sites for vinculin and other actin-regulatory proteins, thereby strengthening and stabilizing the junction.^3^ Force-induced remodeling of AJs involves the coordinated reorganization of E-cadherin nanoclusters, driven by actin polymerization, myosin II contractility, and endocytic recycling of E-cadherin molecules.^2^^,^^6^^,^^7^ Understanding how mechanical and biochemical cues regulate the nanoscale organization and dynamics of E-cadherin clusters is therefore central to understanding how epithelia sense, respond to, and generate mechanical force during tissue morphogenesis.

A fundamental understanding of how mechanical force regulates E-cadherin dynamics and organization at the nanoscale is limited by the diffraction barrier of conventional fluorescence microscopy. Super-resolution DNA-based Point Accumulation for Imaging in Nanoscale Topography (DNA-PAINT) microscopy overcomes this barrier by relying on the transient binding of fluorophores to target proteins using single-stranded DNA (ssDNA) oligonucleotides. A docking strand is attached to a target protein (e.g., via a nanobody), and a complementary imager strand is attached to a fluorophore (e.g., Cy3B). During imaging, the fluorescent probe binds to the target protein and emits a strong fluorescence, or “blink,” lasting for a short time. These blinking or binding events are collected over time and reconstructed to form an image with a spatial resolution of 10-20nm. DNA-PAINT offers unique advantages: it is highly multiplexable, allows quantitative counting of individual molecules, and has been optimized for imaging tissue samples.^8^ DNA-PAINT will enable us to resolve the trans clusters of E-cadherin molecules in fixed tissues. The technique can be exploited to examine the dynamicity and plasticity of AJs in response to mechanical and biochemical cues using mechanically challenged tissues or genetic loss-of-function mutants.

*Drosophila* embryos provide a suitable model system for *in vivo* study of AJ assembly and remodeling.^9^ AJs begin to form during cellularization and mature during gastrulation stage.^10^ During gastrulation, epithelial cells in the lateral epidermis undergo cell intercalation^11^ and provide an excellent model system to understand the dynamic organization, remodeling and cortical anchoring of E-cadherin complexes and will provide mechanistic insights of tissue morphogenesis.

The protocol outlined here describes the collection of embryos at the gastrulation stage, Stage 6 to Stage 8 of development, followed by methanol-free formaldehyde fixation and permeabilization suitable for immunostaining, and mounting of embryos onto chamber slides. Each step has been carefully optimized to preserve the nanostructure of junction complexes and the actomyosin network in the epidermis, enabling imaging at nanometer resolution.

### Innovation

DNA-PAINT is a powerful technique for imaging subcellular structures at nanometer-scale resolution. However, most existing DNA-PAINT protocols have been established in cultured cells, and imaging in tissue remains challenging. Tissues are thick, heterogeneous assemblies of cells that introduce complications including high background fluorescence, poor probe penetrance, and non-specific probe binding.

We have established DNA-PAINT imaging in tissue, specifically in *Drosophila* embryos, to visualize junctional complexes, combining DNA-PAINT nanoscopy with spinning-disk confocal microscopy for optical sectioning. This approach achieves a lateral resolution of approximately 15 nm and an axial resolution of approximately 500 nm several micrometers inside intact tissue, enabling direct visualization of E-cadherin and F-actin nanostructure within native embryonic architecture.

During and after gastrulation in *Drosophila* embryos, E-cadherin organizes and concentrates at the sub-apical pole of lateral ectodermal epithelial cells, forming a narrow junctional belt. We used a transgenic line in which E-cadherin is endogenously GFP-tagged at its C-terminus to image E-cadherin clusters within this belt. We optimized and standardized a fixation and immunostaining protocol for *Drosophila* embryos to improve imager probe penetrance and reduce non-specific nanobody binding, while preserving tissue and plasma membrane integrity.

This protocol uses methanol-free formaldehyde fixation to preserve junctional architecture, with fixation time and concentration optimized to balance antigen preservation with tissue morphology. Permeabilization, blocking, and nanobody incubation/washing steps were optimized to improve signal-to-noise ratio and binding specificity. Samples are mounted on Cell-Tak-coated glass-bottom chambers for stable adhesion during imaging. This protocol can be broadly applied to imaging other GFP- or epitope-tagged proteins via nanobodies in thick or optically challenging tissue. However, optimization will be required as the expression and localization of tagged molecules inside cells might differ.

### Raising flies for egg collection

**Timing: 2–3 days**

**Figure 1:**
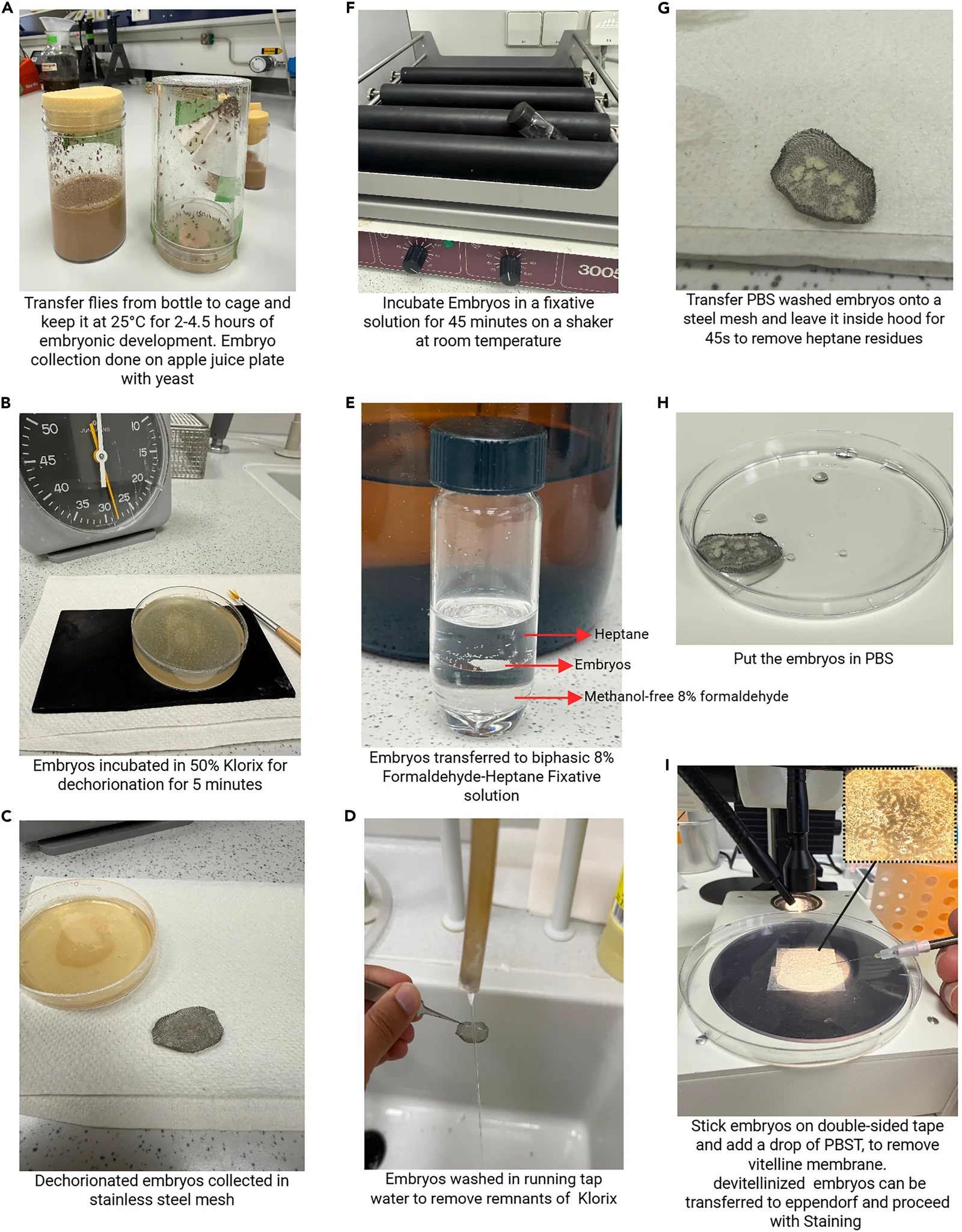
Workflow for embryo collection and fixation Set up fly cage for embryo collection (A). Follow steps (B–D) for dechorionation of embryos. Embryos are transferred to the biphasic fixative solution for fixation and sample preparation (E–H). Manually remove the vitelline membrane from fixed embryos using a fine hypodermic needle (I).
2. Keep the flies at 25°C in a 12 h light: 12 h dark cycle and humidity-controlled incubator for a day or two and change apple juice plates twice or thrice in a day.

***Note:*** This step will acclimatize and synchronize flies for egg collection and embryo staging onto apple juice plates. Collect a large number of embryos to compensate for losses during immunostaining and mounting, as reorienting embryos is quite challenging in the following protocol.

1. Transfer around 100–200 flies into a clean cage and feed them on yeast on apple juice plates (Figure 1A).

## Key resources table

**Table:** 

REAGENT or RESOURCE	SOURCE	IDENTIFIER
**Antibodies**		

MASSIVE-TAG-Q anti-GFP-docking site 3 (1:250)	Massive Photonics	N/A
Imager 3 Cy3B/ATTO 594 (1:1000)	Massive Photonics	N/A
Peptide Cy3B-Lifeact (1:1000)	Peptide Protein Research Ltd	N/A

**Chemicals, peptides, and recombinant proteins**		

Cell-Tak™ Cell and Tissue Adhesive	Corning	Cat#354240
16% Formaldehyde solution (w/v), methanol-free	Thermo Scientific	Cat#28906
Bleach, 6% sodium hypochlorite	DanKlorix	N/A
Bovine serum albumin	Carl Roth	CAS: 9048–46-8
Triton X-100	Carl Roth	CAS: 9002-93-1
Tween 20 (Polysorbate 20)	Carl Roth	CAS: 9005-64-5
Sodium Chloride	Sigma-Aldrich	S9888; CAS:7647-14-5
n-Heptane	Thermo Scientific	CAS: 142-82-5

**Experimental models: Organisms/strains**		

*D. melanogaster*: w; *E-cadherinGFP* (knock-in)	Yang Hong’s Lab	Huang et al.^12^; Gastrulating embryos, Stages 6-8 of development

**Software and algorithms**		

BioRender	BioRender	https://biorender.com
FIJI/ImageJ	Open Source	https://imagej.net/software/fiji

**Other**		

μ-Slide 8 Well Glass Bottom	Ibidi	Cat#80827
Fly cages	Genesee Scientific	Cat#59-100
Flystuff- Mesh basket	Biozol	Art. GNS-46-101
Petri dish	Carl Roth	Art. N224.2
Hypodermic Needle 27G 3/4-inch, 0.4 × 20 mm	Braun	Art. 4657705
Disposable Borosilicate Glass Pasteur Pipets	Carl Roth	Art. 4518
Scintillation vial, 20 mL glass borosilicate	DWK Life Sciences	Cat#W224634
Double-sided adhesive tape	Tesa	Art. 51571

## Materials and equipment

**Table:** 10× PBS

Reagent	Final concentration	Amount
Disodium hydrogen phosphate dihydrate	70 mM	12.46 g
Sodium chloride	1.3 M	76 g
Sodium dihydrogen phosphate monohydrate	30 mM	4.14 g
Milli-Q water	N/A	to 1 L
Total	10× PBS	1 L

Adjust pH to 7.2–7.4. Store at 20°C–22°C for up to several months.

Add 100 mL of 10× PBS to 900 mL of Milli-Q water to make 1× PBS.

Add 100 μL of Triton X-100 to 99.90 mL of 1× PBS to make PBTx.

Add 200 μL of Tween-20 to 99.80 mL of 1× PBS to make PBT.

**Table:** 5% Blocking Buffer

Reagent	Final concentration	Amount
Bovine Serum Albumin (BSA)	5%	5 g
PBT	1×	to 100 mL
Total	5% BSA	100 mL

Store at 4°C for up to 1 month

**Table:** Washing Buffer

Reagent	Final concentration	Amount
Sodium chloride (3M)	300mM	10 mL
10× PBS	1×	10 mL
Tween-20	0.2%	200 μL
Milli-Q water	N/A	to 100 mL
Total	–	100 mL

Store at 20°C-22°C for up to several months.

**Table:** **Fixation solution:** 8% Formaldehyde fixation solution

Reagent	Final concentration	Amount
16% FA (Formaldehyde)	8%	1 mL
2× PBS	1×	1 mL
n-Heptane	N/A	2 mL

Store at 4°C for not more than 48 h.

**CRITICAL:** FA is toxic and can produce fumes. This step has to be performed in the hood wearing gloves.

## Step-by-step method details

### Collection of embryos and fixation

**Timing: 6 h**

1. Collect embryos on an apple juice plate, 2–4.5 h for the stage 6-9 of embryonic development at 25°C (Figure 1A).

***Note:*** Adjust the embryo collection and staging according to the desired embryonic developmental stage.

2. Prepare fresh fixative solution (8% Formaldehyde in PBS) in a scintillation vial. Add the same volume of heptane to the fixative solution.

***Note:*** Formaldehyde solution should be methanol-free. Traces of methanol distort membrane integrity and compromise image quality and resolution.

3. For dechorionation of embryo, cover the apple juice plate with 50% bleach solution, gently brush off the embryos and incubate for 3-5 min (Figure 1B).
4. Collect the embryos on the net (Figure 1C).
5. Wash the embryos with tap water carefully to remove all the remanent of bleach solution (Figure 1D). Dry the embryos in the net on a paper towel.
6. Transfer embryos into the fixative solution and incubate for 45 min on a shaker (Figure 1E and 1F).

**CRITICAL:** If the embryos are not fixed properly, then they will be fragmented during the permeabilization step.

7. Remove the embryos from the interface of a fixative solution to a net and leave it under hood for 45 s to remove all residual heptane (Figure 1G).
8. Wash embryos with PBS to remove traces of formaldehyde solution (Figure 1H).
9. Manually remove the vitelline membrane with a fine hypodermic needle (Figure 1I).
10. Transfer embryos to a 1.5 mL Eppendorf tube.

***Note:*** A pause can be taken here. Embryos can be stored in PBS at 4°C for up to 10 days.

Either pause or proceed to the next step.

### Permeabilization and blocking of sample

**Timing: 2 h**

This section describes the steps to permeabilize embryos for better reagent penetrance and binding specificity.

11. Wash embryos once with PBTx.
12. Incubate embryos in 1 mL volume of PBTx for 1 h at 4°C on the wheel.
13. Wash embryos once with PBT to remove traces of PBTx.
14. Incubate embryos in 1 mL volume of blocking buffer for 1 h in 24°C–26°C on the wheel.

***Note:*** Blocking can be done for 30 min but extending the blocking time from 30 min to 1 h reduced non-specific nanobody binding.

### Nanobody binding

**Timing: 2 h**

This section describes steps for staining GFP- or other epitope-tagged proteins via nanobodies. This section can be skipped if one is only interested in looking at F-actin structure.

15. Remove the blocking buffer and incubate embryos with 500 μL volume of GFP nanobody (20nM in blocking buffer) for 1 h in 24°C–26°C on the wheel.

***Note:*** The nanobody concentration and incubation time may require optimization depending on the expression level and accessibility of the tagged protein of interest. In this study, nanobody incubation was performed for 1 h, as no appreciable difference in image quality was observed upon extending the incubation time to 2 h.

16. Rinse embryos with 1 mL volume of PBT three times to remove residual traces of GFP nanobody in the solution.
17. Wash embryos with 1 mL volume of washing buffer for 15 min for four times over one hour.

### Coating of Cell-tak glue onto chambered slides and mounting of sample

**Timing: 1.5 h**

18. Coating of the chambers with Cell-tak glue.
  a. Mix 47μL of 0.1M sodium bicarbonate with 1 μL of 1M NaOH in 0.5mL Eppendorf tube for each chamber.
  b. Add 2μL of Cell-Tak to raise the final volume to 50 μL.
  c. Immediately mix and pipette the solution onto a slide chamber.
  d. Spread the solution with the pipette tip.
    ***Note:*** Adhesive solution should not be left in the tube for long and should be spread onto the chambered glass bottom as soon as it is made. Storing it long in tube will affect the adhesive quality of the Cell-Tak on the chamber, and embryos will not stick properly to the glass bottom.
  e. Leave the solution in the chamber for 20 min.
  f. Wash three times with 200 μL volume of ddH_2_O.
    ***Note:*** Do not dry out the glue and leave the last wash of ddH_2_O in the chamber until you are ready to mount the sample.
19. Mounting of sample onto the Chamber.
  a. Wash embryos with 1mL volume of PBS three times to remove all detergent.
  b. Remove water from the chamber.
  c. Cut the end off a yellow tip and coat the surface of tip with PBS.
  d. With the cut-off tip, transfer the embryos to the chamber in as little volume as possible.
  e. Using 10 μL pipette tip remove buffer from the chamber and around embryos as much as possible.
    ***Note:*** This step is crucial and should be done with care. If there is a good volume of buffer around embryos, it will interfere with the sticking of embryos on to the chamber. Too much drying of embryos will also lead to embryo damage and rupturing.
  f. Leave the chamber slide with embryos in a humidified chamber for 45 min so that the embryos stick firmly to the glass bottom and do not get dry and burst during the procedure.
  g. After 45 min, rinse embryos first with 200 μL volume of PBS followed by imaging buffer once.
    ***Note:*** If the embryos do not float while adding PBS, embryos are firmly attached to the glass bottom. If not, then collect the embryos floating in PBS and prepare another Cell-Tak patch in the chamber and repeat the mounting step.
  h. Add 200 μL volume of imaging solution with imager probe and close the lid. The slide is now ready for imaging.
    ***Note:*** For single color recording of E-cadherin GFP in lateral epidermis, fill the chambers with 1 nM imager complementary to the nanobody docking strand, labelled with the fluorescent dye Cy3B, prepared in imaging buffer from Maasive Photonics.
    For single color recording of F-actin in lateral epidermis, add 1 nM Cy3B conjugated to the peptide Lifeact in the imaging solution.
    For dual-color recording of E-cadherin and F-actin, add two separate imager probes to the same solution. Use the imager nucleotide for GFP conjugated with Atto594 and the peptide Lifeact conjugated with Cy3B. The final concentration of each imager probe should be adjusted so that the total combined concentration equals that used in single color recording, as doubling the total imager concentration will result in excessive overlapping of blinks and therefore poor image resolution.
    The imager concentration can be optimized in this step; if blinks are overlapping, the concentration can be reduced by diluting the imaging solution.

### Image acquisition

**Timing: 1 ROI = 3.5 h**

Imaging was performed on a custom-built inverted spinning disk microscope configured for SMLM data acquisition. All imaging was carried out with a 100× 1.35 NA silicone immersion objective lens. For a single region of interest, 60,000 unprocessed camera frames were collected in blocks (cycles) of 1,000 frames at a frame rate of 5 Hz. Blink detection, drift correction (real-time and post-processing), and image reconstruction were performed using the established acquisition and processing pipeline.^13^

### Data collection and segmentation

**Timing: 10 min per junction**

**Figure 2:**
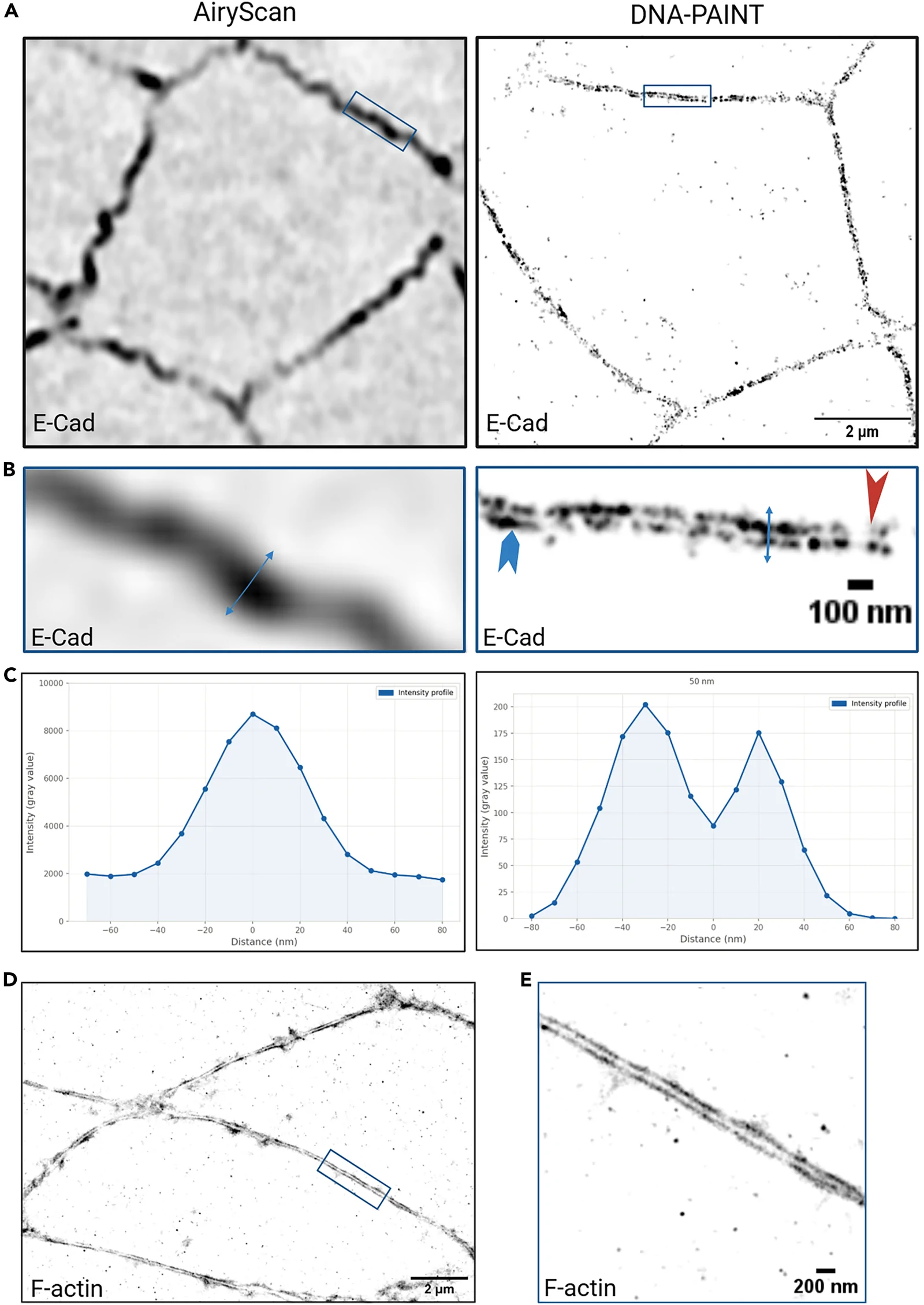
E-Cadherin trans clusters and F-actin structure resolved by DNA/peptide-PAINT microscopy (A) Representative images of E-Cadherin staining from lateral epidermis of Stage 8 embryo of *Drosophila melanogaster* comparing Airyscan and DNA-PAINT microscopy. (B) Magnified view of the junctions as indicated in blue box (A). Trans clusters of E-Cadherin resolved by DNA-PAINT microscopy. Blue arrow indicates paired trans clusters, and red arrow indicates unpaired clusters. (C) Intensity Plot profile along the junction indicated by blue line in (B) The two intensity peaks along junction resolved in DNA-PAINT microscopy. (D) Resolved F-actin structure along the junction by Peptide-PAINT. (E) Magnified view of junction in blue box in (D).

Following image reconstruction,^13^ high-resolution DNA-PAINT nanoscopy images of E-cadherin and F-actin were obtained from *Drosophila* embryos (Figure 2). For downstream quantitative and statistical analyses, image segmentation is required. The two opposing sides of E-cadherin and F-actin within adherens junctions (AJs) are readily resolved in the DNA-PAINT images (Figure 2). To quantify parameters such as cluster size, cluster distribution, pairing of E-cadherin clusters across the junction, and protein colocalization, individual junctions are manually segmented and subsequently straightened. A central midline is then defined to divide each junction into left and right sides, providing the spatial reference for side-specific measurements. Segmentation and straightening are performed using Fiji/ImageJ.

**Figure 3:**
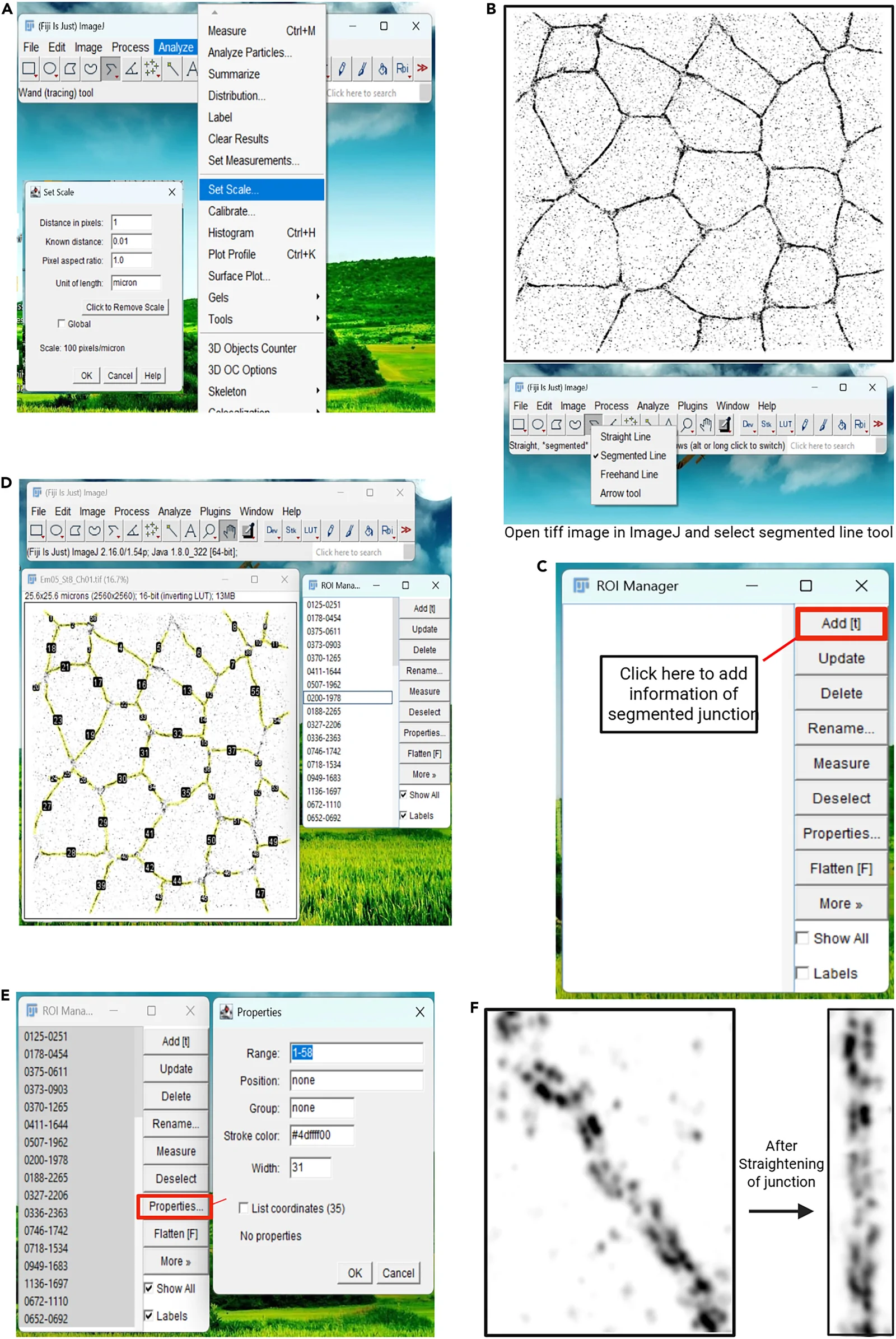
Pipeline for Segmentation and Straightening of junction (A) Set image size in terms of pixels per unit of length. (B) Open tiff image using Fiji or ImageJ and select ‘Segmented Line’ tool to mark the midpoint of the junction. (C) Open ROI manager and add the segmented junction information using the ‘Add[t]’ tab. (D) Segment all the junctions on the image and save their positional information. (E) Select all the segmented junctions and set the width to 31. (F) Curved junction straightened after Straighten function in ImageJ.

20. Set image size (1 pixel = 10 nm in our recording).
  a. Click on Analyze and then Set Scale.
  b. Put in the following information in the dialog box (as shown in Figure 3A):
     1 pixel = 10 nm.
     Distance in pixels: 1.
     Known distance: 0.01.
     Pixel aspect ratio: 1.0.
     Unit of length: micron.
  c. Press “OK” and save.
21. Midline segmentation of junction.
  a. Open the tiff image using Fiji or ImageJ.
  b. Open the ROI Manager via Analyze -> Tools -> ROI Manager.
  c. Right-click to select the “segmented line” tool to mark junction (Figure 3B).
    i. Using the left mouse button, manually select junctions by drawing a polygon along the gap between two lines.
    ii. Use the right mouse button to finish.
  d. Add junction information to ROI Manager:
    Click “Add [t]” in the ROI Manager window (Figure 3C).
    ***Note:*** Repeat this process to mark all junctions on the image and add their information to the ROI Manager.
  e. Save the position information of the segmented junction (Figure 3D).
    i. In the ROI Manager window, click on More -> Save to save the position information.
    ii. Save segmentation data as RoiSet.zip.
22. Junction straightening and saving positional information.
  ***Note:*** A pause can be taken here, and one can load the previously saved segmentation file in the ROI Manager:
  a. Open the image in Fiji or ImageJ.
  b. Open the ROI Manager window via Analyze -> Tools -> ROI Manager.
  c. In the ROI Manager window, load the saved segmentation information by selecting More -> Open and selecting the corresponding RoiSet.zip file.
  d. In the ROI Manager window, check “Show All” and “Labels”.
       The segmentation information will be displayed on the image.
       Or follow from here if continuing after completing step 21:
  e. Select all junctions using “Control + click”, then click “Properties”.
    i. Set Width to 31 (number of pixels, equivalent to 310 nm here)
    ii. Click OK to confirm (Figure 3E).
      ***Note:*** Line width must be chosen in accordance with the junction, and it should cover all the information along the left and right side of the membrane. Width must be at least 21 and an odd number.
  f. Click to select a junction, then go to Edit -> Selection -> Straighten.
  g. Select the straightened image (Figure 3F).
  h. Go to File -> Save As -> PNG and save the straightened image.
    ***Note:*** Naming convention: It should be the same for all junctions in an image and saved in a folder with the same naming convention.
    WT8_eN_001.png = wild type, stage 8, embryo N, junction 1.
    WT8_eN_002.png = wild type, stage 8, embryo N, junction 2.
  i. Extract position information of the junction,
    i. Click to select junction in the ROI manager.
    ii. Click on Edit -> Selection -> Properties.
    iii. Set “List Coordinates” and then Press OK.
  j. Go to File -> Save As and save the positional information in csv excel file.
    ***Note:*** Follow the same naming convention as the straightened image.
    WT8_eN_001.csv = wild type, stage 8, embryo N, junction 1.
    WT8_eN_002.csv = wild type, stage 8, embryo N, junction 2.
    ***Note:*** Position information corresponds one-to-one with the straightened image.

## Expected outcomes

Following this protocol, *Drosophila* embryos should be well-preserved with intact membrane architecture, suitable for high-resolution DNA-PAINT nanoscopy of E-cadherin and F-actin structure. The use of methanol-free formaldehyde is critical at the fixation step as traces of methanol may compromise the structural integrity of membrane-associated proteins and lead to artifactual redistribution of E-cadherin at cell-cell junctions. Methanol-free formaldehyde ensures gentle crosslinking that preserves the native membrane bilayer and the spatial organization of transmembrane protein complexes.

Upon successful fixation and staining, the cellular architecture of E-cadherin should be well-preserved, with junctional E-cadherin localizing in a continuous belt-like pattern within which trans clusters should be resolvable, as shown in Figures 2A and 2B. F-actin should similarly display intact cortical organization without signs of collapse or aggregation.

DNA-PAINT imaging of correctly processed embryos should provide resolution below 40 nm, allowing visualization of the GFP-tagged distal ends of E-cadherin trans-clusters across opposing membranes. At this resolution, individual trans-clusters should be distinguishable as discrete nanoscale assemblies, providing direct structural evidence of E-cadherin-mediated intercellular adhesion at the molecular level.

To assess labelling specificity, embryos lacking the GFP-tagged target should be processed under identical conditions as a no-GFP control. Junctional signal should be absent or negligible in this control, confirming that the observed localization reflects specific nanobody binding rather than non-specific tissue interactions. The mitotic mosaic recombination system can also be used to assess GFP-nanobody labeling specificity; mosaic clones of GFP-expressing cells surrounded by GFP-negative neighbors provide an internal control for labeling specificity within the same tissue, under identical fixation and staining conditions.^14^ Omission of the imager strand serves as an additional control for non-specific docking strand binding and should yield no detectable blink events above background.

Failure to achieve this resolution typically indicates suboptimal fixation, excessive background from non-specific docking strand binding, or insufficient imaging buffer performance. In such cases, the troubleshooting section should be consulted.

After image reconstruction, junctions are segmented and straightened using Fiji or ImageJ. This segmentation and straightening section of the protocol will provide junctional information suitable for quantitative correlation analysis.

## Limitations

DNA-PAINT, like all fluorescence-based super-resolution techniques, relies on the assumption that labelling efficiency is uniform across the sample. However, labelling efficiency is rarely homogenous and it can vary due to local differences in probe accessibility, epitope density, and fixation quality. In a dense cellular environment, antibodies or nanobodies may not reach all target proteins, leading to underrepresentation of the true molecular distribution. Users should therefore interpret quantitative measurements such as cluster density or molecular spacing with this limitation in mind.

Nanobodies are more advantageous than conventional antibodies in super-resolution nanoscopy owing to their small size, which enables better access to densely expressed antigens. However, in the context of E-cadherin imaging, nanobody accessibility can be compromised by the dense packing of adhesion complexes at cell-cell junctions. Additionally, nanobody binding efficiency is sensitive to fixation conditions; over-fixation can mask the GFP epitope and reduce nanobody accessibility, while under-fixation may compromise structural preservation.

Lifeact is a peptide probe used for labelling F-actin in fixed and live samples. In fixed samples, Lifeact may not label all F-actin structures with equal efficiency, and its binding can be influenced by the degree of crosslinking introduced during fixation. Lifeact does not differentiate between actin filament populations, such as linear and branched F-actin, which limits the specificity of structural interpretations derived from Lifeact-based imaging. This should be taken into account when drawing conclusions about cortical actin architecture at epithelial junctions.

## Troubleshooting

### Problem 1

Poor Fixation and fragmentation of embryo (Step 6).

### Potential solution

Optimize fixation time and shaker speed to allow the fixative to penetrate the membrane and cross-link proteins. Vigorous shaking of the heptane-formaldehyde biphasic solution will allow tiny droplets of formaldehyde to enter the hydrophobic vitelline membrane along with heptane and cross-link the whole-mount embryo. Fixation time can vary according to the embryonic developmental stage; gastrulating embryos can be fixed for 45-60 min on an orbital shaker.

Triton X-100 detergent is harsh on the embryos, so the permeabilization with 0.1% TritonX-100 should be done at 4°C. If embryos are not fixed properly, they will fragment in this step, which is an indicator of insufficient fixation. In such cases, increase the fixation time or shaker speed, change only one parameter at a time: first increase the orbital shaker speed, taking care not to agitate too vigorously as excessive agitation may damage the embryos. If fragmentation persists, increase the fixation time, noting that excessive fixation time may reduce probe accessibility and raise background signal.

### Problem 2

Embryos stuck on the Eppendorf surface or in tips when suspended in PBS (Step 19).

### Potential solution

It may happen due to remains of the vitelline membrane present on the embryos. Hand-peeling of the vitelline membrane should be done properly. One may also take a higher number of embryos in the beginning, so in the end you have enough left for imaging.

### Problem 3

Embryos do not firmly attach to the glass bottom (Step 19).

### Potential solution

While mounting samples, avoid overcrowding embryos in a chamber. Try to remove PBS from the chamber as much as possible. One may also increase and optimize the mounting time in the humidified chamber to ensure proper sticking of the embryo with little damage to the sample.

### Problem 4

Poor number of blinks during recording of images.

### Potential solution

Permeabilization step (Step 12) with Triton X-100 not done properly, and one may increase the permeabilization time to improve probe accessibility and blink frequency.

### Problem 5

High background noise in the sample.

### Potential solution

During the washing step (Step 17), one may add a higher concentration of NaCl, 300-500mM, to get rid of non-specific binding of nanobodies. Also, imager concentration needs to be optimized to reduce signal-to- noise ratio and will eventually improve the resolution and quality of image.

## Resource availability

### Lead contact

Further information and requests for resources and reagents should be directed to and will be fulfilled by the lead contact, Deqing Kong (deqing.kong@ouc.edu.cn)*.*

### Technical contact

Technical questions on executing this protocol should be directed to and will be answered by the technical contact, Neelima Sharma (neelima.sharma@biologie.uni-marburg.de).

### Materials availability

This study did not generate new unique reagents.

### Data and code availability

This study did not generate or analyze any new data or code.

## Acknowledgments

This work was supported by 10.13039/501100001659Deutsche Forschungsgemeinschaft (10.13039/501100001659DFG GR1945/15–1, GR1945/17-1) and the 10.13039/501100007129Shandong Provincial Natural Science Foundation, China (2026HWYQ-023). We also thank the service support from the Bloomington Drosophila Stock Center (supported by NIH P40OD018537). Sincere gratitude to The Boehringer Ingelheim Fonds (10.13039/501100001645BIF) travel grant for a short-term research stay in Cambridge for performing experiments. The graphical abstract and figures were created using BioRender.com.

## Author contributions

J.G. and D.K. established and standardized the experimental procedure in embryos. M.H. established the segmentation procedure. J.H.R. and D.St J. established the experimental procedure. N.S., J.G., and D.K. performed the experiments. N.S. wrote the manuscript with comments from J.G. and D.K.

## Declaration of interests

The authors declare no competing interests.
